# Arts Syndrome and Involuntary Eye Movements

**DOI:** 10.1002/mdc3.70608

**Published:** 2026-03-27

**Authors:** Victor Rebelo Procaci, Victor Augusto Vanderlinde Camara, Flavio Moura Rezende Filho, José Luiz Pedroso, Orlando Graziani Povoas Barsottini

**Affiliations:** ^1^ Department of Neurology Universidade Federal de São Paulo São Paulo Brazil

**Keywords:** arts syndrome, nystagmus, opsoclonus, PRPS1

## Question

**Figure 1:**
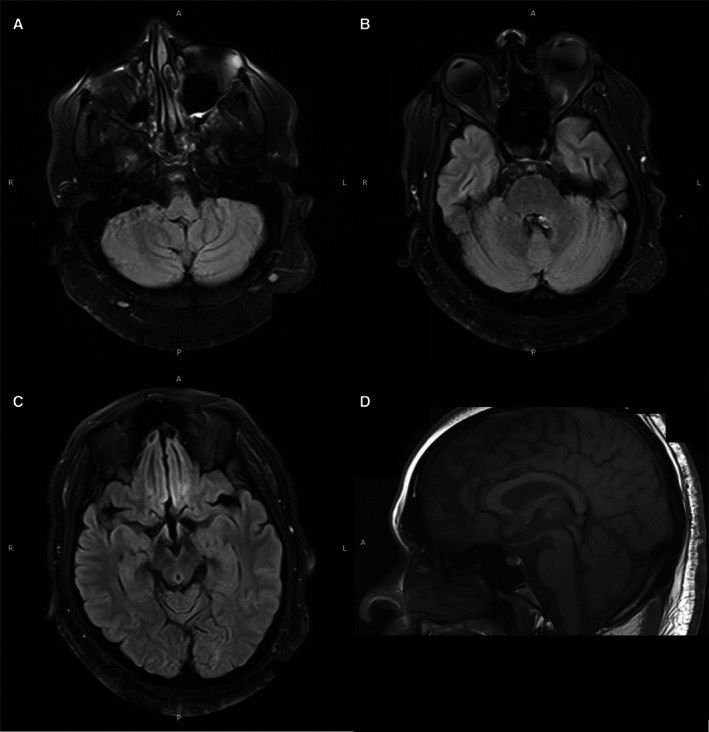
Axial FLAIR (A–C) and sagittal T1‐weighted (D) brain MRI demonstrating no structural abnormalities.

A 38‐year‐old man with genetically confirmed Arts syndrome, *PRPS1*:c.706C>T; p.(Leu236Phe), was referred for evaluation of involuntary eye movements (Video 1). His neurological phenotype included congenital sensorineural deafness, optic neuropathy with severe visual impairment, ataxia, and peripheral neuropathy. Brain MRI was unremarkable (Fig. 1).

**Video 1:** Video Part 1. Recording at normal speed shows continuous, multidirectional involuntary eye movements, with impaired fixation and no evidence of gaze palsy. Video Part 2. Slow‐motion recording facilitates characterization of the eye movements, allowing visualization of continuous, smooth elliptical oscillations of the eyes. The oscillation follows a counterclockwise elliptical trajectory when viewed from the examiner's perspective, consistent with the temporal sequence of eye positions and a phase relationship in which one component leads the other. This sequence is best appreciated between 1:19 and 1:28.

**What is the eye movement disorder shown in the video and the most appropriate treatment?**

1. Opsoclonus—corticosteroid pulse therapy
2. Square‐wave jerks—optical rehabilitation
3. Elliptical pendular nystagmus—gabapentin
4. Functional eye movement disorder—cognitive behavioral therapy

## Answer

The patient exhibits elliptical pendular nystagmus, a form of acquired pendular nystagmus in which the eyes oscillate along an elliptical trajectory, reflecting the vectorial summation of simultaneous horizontal and vertical oscillatory components. The geometric form of pendular nystagmus depends on the relative amplitudes and phase relationships between synchronized oscillations in different planes. Circular trajectories occur when horizontal and vertical components are 90° out of phase with equal amplitudes, whereas elliptical trajectories arise when amplitudes differ and when the phase relationship deviates from 90° (Supplementary Appendix S1). The underlying pathophysiology is attributed to dysfunction of the gaze‐holding neural integrator network and its cerebellar modulation, involving the medial vestibular nucleus and nucleus prepositus hypoglossi for horizontal gaze, the interstitial nucleus of Cajal for vertical gaze, and their feedback connections with the cerebellar flocculus and paraflocculus. In this patient, severe visual loss likely contributed to destabilization of neural integrator calibration, a recognized mechanism for acquired pendular nystagmus.^1^ Pendular nystagmus has been previously reported in Arts syndrome.^2^

Elliptical nystagmus must be distinguished from opsoclonus, which consists of chaotic, high‐frequency, multidirectional back‐to‐back saccades without intersaccadic intervals, reflecting dysfunction of saccadic burst and omnipause neuron control rather than gaze‐holding failure. Unlike opsoclonus, elliptical nystagmus shows smooth sinusoidal slow phases and a regular geometric trajectory. Gabapentin may be used to reduce nystagmus intensity and alleviate oscillopsia.^1^

## Author Roles

(1) Research project: A. Conception, B. Organization, C. Execution; (2) Statistical Analysis: A. Design, B. Execution, C. Review and Critique; (3) Manuscript: A. Writing of the first draft, B. Review and Critique.

V.R.P.: 1A, 1B, 1C, 3A.

V.A.V.C.: 1B, 3B.

F.M.R.F.: 1B, 3B.

J.L.P.: 1A, 1B, 3B.

O.G.P.B.: 1A, 3B.

## Disclosures

**Ethical compliance statement:** This study was approved by our local Ethics Institution. Patient consent form was obtained. We confirm that we have read the Journal's position on issues involved in ethical publication and affirm that this work is consistent with those guidelines.

**Funding Sources and Conflict of Interest:** No specific funding was received for this work. The authors declare that there are no conflicts of interest relevant to this work.

**Financial disclosures for the previous 12 months:** VRP has received honoraria from Biogen for delivering educational lectures within the past 12 months.

## Financial Disclosures and Conflicts of Interest

Author disclosures are available in the Supporting Information.

## Supporting information


**Appendix S1.** Supporting references related to the pathophysiological mechanisms of pendular nystagmus.

**Data S1.** Supporting Information.

## Data Availability

Data sharing not applicable to this article as no datasets were generated or analyzed during the current study.
