# Supplementary material for: Arts Syndrome and Involuntary Eye Movements
Source: Mov Disord Clin Pract. 2026 Mar 27:10.1002/mdc3.70608. Online ahead of print. doi: 10.1002/mdc3.70608 (PMC13338961; doi:10.1002/mdc3.70608)
Supplement: Supplementary file 2 — Appendix S1. Supporting references related to the pathophysiological mechanisms of pendular nystagmus. [file MDC3-9999-0-s001.docx]

**SUPPLEMENTARY APPENDIX**

Additional supporting references related to the pathophysiological mechanisms of pendular nystagmus:

1. Rabinovitch HE, Sharpe JA, Sylvester TO. The ocular tilt reaction. A paroxysmal dyskinesia associated with elliptical nystagmus. Arch Ophthalmol. 1977 Aug;95(8):1395-8. doi: 10.1001/archopht.1977.04450080105012. PMID: 889516.
2. Lopez LI, Bronstein AM, Gresty MA, Du Boulay EP, Rudge P. Clinical and MRI correlates in 27 patients with acquired pendular nystagmus. Brain. 1996 Apr;119 ( Pt 2):465-72. doi: 10.1093/brain/119.2.465. PMID: 8800942.
3. Kang S, Shaikh AG. Acquired pendular nystagmus. J Neurol Sci. 2017 Apr 15;375:8-17. doi: 10.1016/j.jns.2017.01.033. Epub 2017 Jan 10. PMID: 28320194; PMCID: PMC5363284.
